# Galea spixii embryos have potential to produce steroid hormones

**DOI:** 10.1590/1984-3143-AR2022-0091

**Published:** 2023-01-16

**Authors:** Franceliusa Delys de Oliveira, Paulo Ramos da Silva Santos, Moacir Franco de Oliveira, Antônio Chaves de Assis

**Affiliations:** 1 Departamento de Cirurgia, Faculdade de Medicina Veterinária e Zootecnia, Universidade de São Paulo, São Paulo, SP, Brasil; 2 Departamento de Ciência Animal, Universidade Federal Rural do Semi-Árido, Mossoró, RN, Brasil

**Keywords:** aromatase, cytochrome P450, concepty, gestation, Spix yellow-toothed cavy

## Abstract

Estrogens and progestogens are hormones produced by maternal organs and it is required for recognition and maintenance of pregnancy. In addition, the embryo may also be a source. For this, the aim was to identify steroidogenic expression on Galea spixii embryos early in the embryonic period. Embryos were collected on Days 10 and 15 of gestation; some were fixed in 4% paraformaldehyde for morphological and immunohistochemical analysis (P450arom), whereas others had RNA extracted to determine presence of CYP19a1 gene. In addition, for immunochemistry, maternal ovaries were collected as positive control tissues. Maternal tissues had positive staining for aromatase, whereas none of the embryos stained for P450 aromatase. Based on qPCR reactions, CYP19a1 gene were expressed in all embryos. Galea spixii embryos expressed steroidogenic genes during the post-implantation period, indicating they have the potential to produce steroid hormones.

## Introduction

The Galea spixii, a wild rodent belonging to Caviidea, Caviomorpha, and Rodentia groups, is widely distributed in Latin America. Morphological characteristics include an elongated body (22.5-23.5 cm) and body weight of 375-405 grams, intermediate between the Muridae and Dasyproctidae groups (which are smaller and larger, respectively) (Schimming et al., 2021; Praxedes et al., 2018). They are covered with hair, ranging from white (ventral region) to yellowish-gray (the dorsal region). They have three digits on their front feet, four on their rear feet and essentially no tail (Oliveira et al., 2010; Percequillo et al., 2007). In addition to being the most abundant mammal in the Caatinga region of Brazil, they are an important source of animal protein for many people (Queiroz et al., 2020), as well as a potential model for reproductive disordes (Arroyo et al., 2021; Santos et al., 2017a, b; Oliveira et al., 2008).

**Figure 1:**
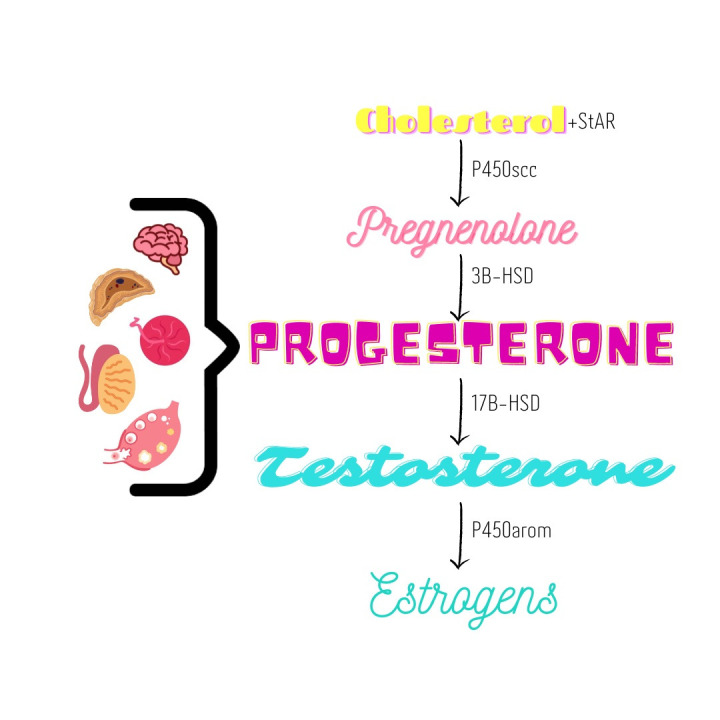
Simplified steroid chain diagram. The cytochrome P450 aromatase is the enzyme responsible for estrogen biosynthesis from androgens.

Steroid hormones are produced by gonads and other organs or tissues, including adrenals, brain, liver, skin, adipose tissue and placenta (Aguilera et al., 2022; Bondesson et al., 2015; Pezzi et al., 2003). The first step in formation of steroid hormones, conversion of cholesterol to pregnenolone, involves cholesterol side chain cleavage by side chain cleavage cytochrome P450 (cytochrome P450scc) (Figure 1). This reaction occurs in the mitochondrial cristae; cholesterol is transported into that location by steroidogenic acute regulatory protein - StAR (Ben-Zimra et al., 2002; Black et al., 1994). The second reaction occurs in the endoplasmic reticulum, where pregnenolone is converted to progesterone by 3β-hydroxysteroid dehydrogenase (3β-HSD) (Ben-Zimra et al., 2002; Borland et al., 1977). Conversion of progestogens to androgens involves cytochrome P450 17α-hydroxylase/17,20-lyase enzyme complex (P450c17). The enzyme 17α-hydroxylase converts pregnenolone to 17α-hydroxypregnenolone, which is converted to dehydroepiandrosterone by C17,20-lyase. Conversion of androgens to estrogens requires steroid hydroxylases, including cytochrome P450 aromatase (P450arom) and the NADPH cytochrome P450 reductase (reductase). These enzymes are encoded by genes CYP (CYP11 - P450scc, CYP17 - P450c17 and, CYP19 - P450arom), which are highly conserved in all vertebrates (Schuler et al., 2006; Ben-Zimra et al., 2002; Conley and Hinshelwood, 2001).

Implantation requires a hatched blastocyst and a receptive uterus. Furthermore, estrogens and progestogens are required for recognition and maintenance of pregnancy (Aguilera et al., 2022; Bondesson et al., 2015; Zhang and Murphy, 2014; Dickmann, 1979). These hormones are produced by maternal organs. In addition, the embryo may also be a source (Levasseur, 1984; Grube et al., 1978; Antila et al., 1977), although false negative results are common, due to inadequate sensitivity of detection methods (Bleau, 1981; Evans and Kennedy, 1980; Angle and Mead, 1979; Singh and Booth, 1979; Grube et al., 1978; Singh and Booth, 1978). The aim of this study was to determine the potential for G. spixii embryos to produce estrogens during the embryonic period.

## Methods

### Ethical aspects

This study was approved and performed under the guidelines of the Ethics Committee for Animal Use of the School of Veterinary Medicine and Animal Science of University of São Paulo (CEUA 2923/2013).

### Sample collection and Study design

The experiment was conducted in the Center for Multiplication of Wild Animals (CEMAS) in the Federal University Rural of Semi-arid (UFERSA-Mossoró/RN/Brazil). Spix yellow-toothed cavy in reproductive ages were placed in boxes, at the ratio of 2 females to 1 male, with water and feed available ad libitum. Vaginal cytology was performed once daily in females. The day on which sperm were detected in the female genital tract was considered the day of mating (D0) and the next day as the first day of pregnancy (Day 1). Thus, to obtain the samples in the days 10 and 15 of pregnancy, the females were anesthetized by intramuscular injection of 0.5 ml of 2% xylazine hydrochloride and 0.5 ml of 10% ketamine hydrochloride and subsequently euthanized with an intracardiac injection of 1 ml of potassium chloride. The uterus was removed and sectioned in regions that contained concepts.

Four conceptuses (n=4) were obtained in the present study. Of these, two conceptuses belonged to day 10 (D10) and the other two were obtained on day 15 (D15). Immediately after obtaining, the conceptuses were submitted to macroscopic and histological evaluation. In addition, the immunostaining profile and gene expression of cytochrome P450 aromatase were also evaluated.

### Macroscopic analysis

The macroscopic characteristics of the conceptuses were subjectively evaluated using a stereomicroscope (Zeiss, Stemi SV6, Germany). Therefore, the uteruses were examined and the implantation sites were considered, as well as the uniformity and size of the conceptuses, corresponding to the respective gestational ages (10 and 15 days).

### Microscopic analysis

Conceptus samples were fixed in 4% paraformaldehyde (Sigma-Aldrich, St. Louis, MO, USA) for 24 h, dehydrated in dilutions (70%-100%) of ethanol (Sigma-Aldrich), and embedded in paraffin (Tolosa et al., 2003). Sections of 5 μm were cut and mounted. Sections were stained using hematoxylin and eosin (Sigma-Aldrich) to investigate blastocyst, surrounding endometrium and embryonic layers formation morphology. Following deparaffinization in xylol (Sigma-Aldrich, Wicklow, Ireland) at room temperature (RT) using two solutions for 10 min each, rehydration in a descending series of etanol concentrations was performed (100%, 2 × 5 min; 5 min each for 90%, 80%, and 70%), followed by distilled water (RT, 5 min). The sections were then stained with hematoxylin for 30 s at RT, washed in running water for 10 min, stained with eosin at RT for 15 s, and washed in running water for 10 min. The slides were then dehydrated in increasing dilutions of ethanol (RT, 5 min 70%, 5 min 80%, 5 min 90%, 2 × 5 min 100%), cleaned in xylene (Sigma-Aldrich) at RT, 2 × 10 min, and mounted on Permount® (SP15-500; Thermo Fisher Scientific, Waltham, MA, USA). The analyses were performed by light microscopy (Nikon Eclipse 80i, Tóquio, Japão).

### Immunohistochemistry

Fixed tissues were deparaffinized and antigen retrieval was performed using a citrate-based buffer (pH 6.0 with detergent, Antigen Retrieval Buffer, PMB1-125, SpringBio), and endogenous peroxidases were quenched with 0.3% H2O2 in methanol for 15 min. After rinsing in phosphate buffered saline (PBS), slides were blocked with ProteinBLock (DPB-125, SpringBio) for 20 min at room temperature. The sections were then incubated in humidified chambers overnight at 4-8°C with the following primary antisera: anti-P450arom (1:50, rabbit polyclonal, ab18995, Abcam). Antisera were diluted in PBS prior to immunolabeling. After incubation with the primary antisera, slides were rinsed for 5 min in PBS and incubated with N-Histofine Simple Stain Mouse MAX PO (Nichirei Biosciences) for 30 min prior to detection using the DAB kit (DAB-125, SpringBio). For negative control, the primray antisera were replaced by PBS, and, for positive control, we followed other aromatase studies (Conley et al., 1996). Slides were then counterstained with hematoxylin and mounted in permount (FISH-SP15-500, Thermo Fisher Scientific).

### Real-time PCR

Tissue samples of conceptus were immersed in RNAlater Stabilization Solution (Ambion, Carlsbad, CA, USA), transported to the laboratory and then stored at −80°C until processing for detection using quantitative reverse transcription PCR (RT-qPCR).

Samples were homogenized on ice in TRIzol Reagent (Ambion, Carlsbad, CA, USA) and total RNA from all testes was extracted according to the manufacturer’s instructions. Total RNA was quantified in all samples using a NanoDrop 2000c Spectrophotometer (Thermo Scientific). All preparations had RNA concentrations >300 ng/μL. RNA was stored at −80°C and used for real-time qPCR.

For RT-qPCR, amplification was performed using a High-Capacity cDNA Reverse Transcription Kit (Applied Biosystems, Carlsbad, CA, USA). Based on information of mouse and guinea pig in NCBI GenBank, G. spixii primers and probes were designed using Primer Express, ver. 1.5 (Applied Biosystems, Carlsbad, CA, USA). All primers and probes were synthesized by Sigma-Aldrich and were used at a concentration of 10 mM. Primer sequences and corresponding base sites are listed in Table 1. For aromatase (Cyp19a1): >gi | 156139071 | ref | NM_007810.3 | Mus musculus cytochrome P450, family 19, subfamily a, polypeptide 1 (Cyp19a1), mRNA; >gi | 1039792035 | ref | XM_006510805.3 | PREDICTED: Mus musculus cytochrome P450, family 19, subfamily a, polypeptide 1 (Cyp19a1), transcript variant X1, mRNA; >gi | 1039792036 | ref | XM_006510806.3 | PREDICTED: Mus musculus cytochrome P450, family 19, subfamily a, polypeptide 1 (Cyp19a1), transcript variant X2, mRNA; >gi | 1039792037 | ref | XM_017313123.1 | PREDICTED: Mus musculus cytochrome P450, family 19, subfamily a, polypeptide 1 (Cyp19a1), transcript variant X3, mRNA; >gi | 1039792039 | ref | XM_006510809.3 | PREDICTED: Mus musculus cytochrome P450, family 19, subfamily a, polypeptide 1 (Cyp19a1), transcript variant X4, mRNA; >gi | 1039792040 | ref | XM_006510808.3 | PREDICTED: Mus musculus cytochrome P450, family 19, subfamily a, polypeptide 1 (Cyp19a1), transcript variant X5, mRNA; >gi | 1039792041 | ref | XM_011242661.2 | PREDICTED: Mus musculus cytochrome P450, family 19, subfamily a, polypeptide 1 (Cyp19a1), transcript variant X6, mRNA. Thermal cycling conditions were as follows: holding stage at 95°C for 15 min, followed by cycling stage, 40 cycles of 10 s at 95°C and 30 s at 63°C, and finally, melting curve stage, 10 s at 95°C, 30 s at 63°C and 10 s at 95°C, were performed in SetpOnePlus Real-Time Systems (Applied Biosystems) using a 96-well optical reaction plate. Each sample was run in triplicate. Gene expression levels were recorded as quantification cycle values and then analyzed using the ΔΔCt method. RT-qPCR data were normalized with glyceraldehyde-3-phosphate dehydrogenase (GAPDH) and 18S (rRNA).

**Table 1:** Primer sequences and probes used for real-time quantitative polymerase chain reaction, along with efficiency and correlation.

	**Accession no.**	**Forward primer (5′-3′)**	**Reverse primer (5′-3′)**	**R^2^**
*CYP19a1*	NM_007810.3	TGGTGGAAGTTTGTGTGGAG	GATGTTTGGTTTGATGAGGAGA	1.00
*GAPDH*	KM_008084	CCAGAACATCATCCCTGCAT	GTTCAGCTCTGGGATGACCTT	1.00
*18S*	V01270	TACCACATCCAAGGAAGGCAGCA	TGGAATTACCGCGGCTGCTGGCA	1.00

CYP19A1 (P450arom); GAPDH (glyceraldehyde-3-phosphate dehydrogenase); 18S (rRNA).

## Results

### Macro and microscopic aspects of the concepts

**Figure 2:**
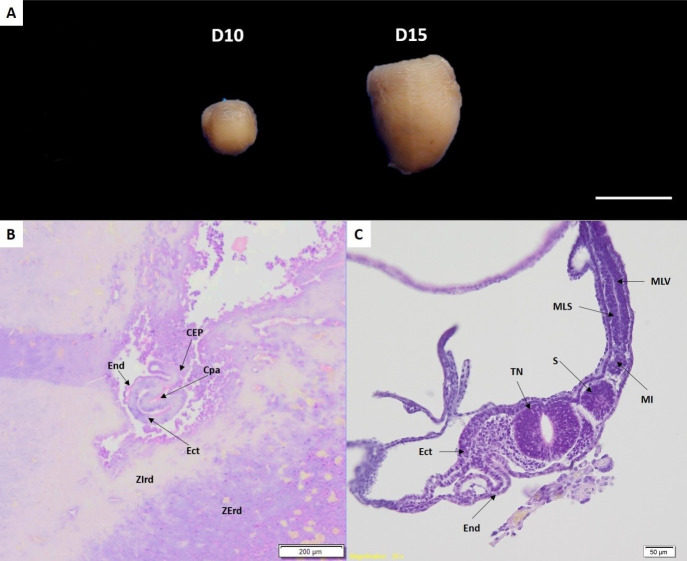
Macro and microscopy of G. spixii concepts. In A, concepts are excised from the uterus (scale bar: 1 cm). In B, microscopy of Day 10 embryo. End - endoderm; CEP - ectoplacental cone; Cpa - proaminiotic cavity; Ect - ectoderm; Zird - inner decidual reaction zone; Zerd - outer decidual reaction zone. In C, microscopy of D15 embryo. TN - neural tube; S - somite; MLS - somatic mesoderm layer; MLV - splanchnic mesoderm layer; MI - intermediate mesoderm. Photomicrography, LM method, (B) bar = 200 μm; (C) bar= 50 μm.

After the uterus was excised, concepts were identified, consistent with gestational age. A large increase were observed of conceptus from Day 10 to Day 15, due to rapid development of the embryo and fetal membranes (Figure 2A). Microscopically, the Day-10 embryo was located near the uterine lumen linked to endometrium by the ectoplacental cone. In the surrounding endometrium, a deciduous reaction (both outside and inner zones) was visible. The cylindrical egg or blasctocist, had a pro-amniotic cavity that formed an invagination in the embryonic ectoderm. Around these cells, there was a layer of embryonic endodermic cells continuous with extra-embryonic endoderm cells (Figure 2B). At this stage, it was not possible to identify formation of mesodermic cells, indicating that at Day 10, the embryo had not reached gastrulation, when the three embryonic layers are formed.

On Day 15, embryonic membranes were well developed. The yolk sac was intimately associated with the uterine epithelium and presented a main layer composed of visceral endoderm. The process of yolk sac reversal had already occurred, based on the absence of omphalopleura, a juxtaposed tissue between the visceral endoderm and uterus (Figure 2C). Consequently, the endoderm of the yolk sac was in direct contact with the endometrium, facilitating exchange between maternal and fetal tissues, prior to development of the definitive placenta. The embryonic disc was at a trilaminar stage, with three embryonic layers and first differentiations. The neural tube was fully formed, including a neural canal. Lateral to the neural tube, the mesoderm intraembryonic was differentiated in three parts: the paraxial mesoderm (somites), intermediary mesoderm, and lateral mesoderm. The latter had two distinct layers, one in contact with the ectoderm (somatic lateral mesoderm) and the other in contact with the endoderm (visceral lateral mesoderm; Figure 2C).

### Cytochrome P450 aromatase immunoreaction and CYP19a1 expression

**Figure 3:**
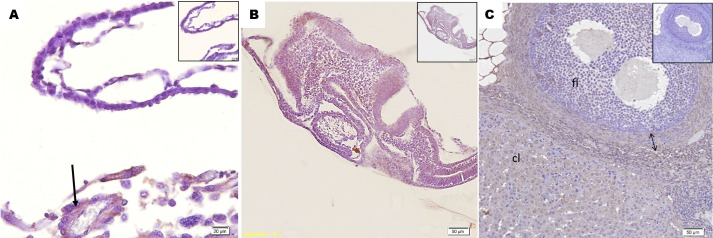
Immunohistochemical expression of cytochrome P450arom in Day15. In A, arrow indicates positive staining in the endometrium. In B, no positive staining on embryo and fetal membranes. In C, note positive staining in ovaries, ovarian follicle theca (double headed arrow), follicle (fl) and corpus luteum (cl). Inserts in micrographs represent negative controls. Photomicrography, IHC method, (A) bar = 20 μm; (B/C) bar= 50 μm.

The P450 aromatase immunoreaction was positive on the Day-15 conceptus in endometrium, adjacent to the yolk sac. This expression was evident mainly next to small blood vessels (Figure 3A). However, none of the embryonic tissues, embryonic membranes or embryonic disc was expressed for P450 aromatase (Figure 3B). The ovarian, maternal tissue and positive control, were positive for P450arom, specifically, in corpus luteum, and ovarian follicle theca (Figure 3C). Based on averages for each ∆CT sample, the CYP19a1 gene expression was detected and increased from Day 10 to Day 15, by RT-qPCR (Table 2).

**Table 2:** ΔCt average for CYP19a1 gene of conceptus (days) detection.

**ΔCt Average**	**Day 10**	**Day 15**
*CYP19a1*	7.877	15.515

## Discussion

In each pregnancy, just one embryo had implanted, although one Day-15 female had three implantation sites. On average, there are 2 to 4 embryos per pregnancy for this species (Oliveira et al., 2008, 2012); therefore, perhaps captivity and interbreeding reduced litter size. Embryonic buttons on Day 15 were much larger on Day 15, indicating rapid development. Although the embryonic disc grows, differentiation of embryonic membranes to initial formation of the placenta contributes even more to this rapid development (Vale et al., 2013).

The embryo morphology of Day-10 G. spixii corresponded to Day 5.5 in rats and mice. We inferred that initial development was slower, due to longer gestation (Kaufman and Kaufman, 1992). On Day 15, embryonic membranes were already well developed and reversal of the yolk sac, which typically occurs in rodents, had already occurred (it was reported to occur in these species on Day 14 (Vale et al., 2013).

Staining for P450arom in maternal tissues studied has been reported (Murakami et al., 2018; Payne and Hales, 2004; Meduri et al., 1996). We inferred that the ovary, adrenal cortex and liver were involved in estradiol production. Although the main site of estrogen production is the female gonad (Bondesson et al., 2015), based on the presence of P450arom in liver and adrenal cortex, these two sites may also have a role. In humans, estradiol production depends on joint activities of placenta, liver, and ovary (Norman and Litwack, 1997). The ovaries are the main sources of progestagens and estrogens (Bulun, 2014; Ben-Zimra et al., 2002). In our study, unlike previous reports, there was positive staining of P450arom in the cells of the corpus luteum and follicular theca. Theca cells produce estrogen, in response to the stimulus of follicle stimulating hormone (FSH), during the estrous cycle for development and ovulation of ovarian follicles. If pregnancy ensues, follicular development is suppressed (Hyttel et al., 2010; McGeady et al., 2006). Perhaps positive staining in theca of pregnant females indicates that this site of maternal hormone production is necessary to support pregnancy.

There were no positive reactions for immunohistochemistry, including embryonic disc and annexes. Trophectoderm involved in formation of embryonic tissues attachments was reported to have steroidogenic capacity (Conley and Hinshelwood, 2001; Walters et al., 2000; Chu et al., 1999), although this capacity may be lost as trophectoderm differentiates. Although there are apparently no reports that endometrial cells have capacity for estrogen formation, estrogens are released locally at the implantation site to initiate an inflammatory process required for the implantation (Dickmann, 1979).

It is known that rodent placentas secrete small amounts of progester and testosterone, and the non-expression of P450 arom, cannot synthesize estrogens (Sun et al., 2012). Whit it, gene expression of enzymes of the P450 complex in G. spixii was interpreted as steroid production by embryos being important during early pregnancy. For example, progesterone and estrogens are required for cleavage and blastocyst differentiation (Zhang and Murphy, 2014; Holdas, 1993). Production of steroid hormones by the fetus has been reported, but the studies used methods that were not highly sensitive (Raeside et al., 2012; Dey et al., 1976; Salomon and Sherman, 1975). Current approaches detect gene expression of enzymes involved in steroidogenesis in various species, similar to what was used in the present study (Zhang and Murphy, 2014; Pezzi et al., 2003; Chu et al., 1999; Choi et al., 1997; Conley et al., 1992).

Progestogens are essential for maintenance of pregnancy and estrogens signal the presence of the embryo to the maternal unit. Genes required to produce these hormones are highly expressed in fetal endocrine organs (e.g. fetal gonads) during sexual differentiation (Greco and Payne, 1994). At this stage of development, expression of these genes are related to development of body features and pregnancy maintenance. This difference of enzyme expression and function occurs due to variations in specific isoforms that are expressed in each period. For example, the CYP19 isoform expressed during implantation differs from that expressed during the formation of the placenta (Choi et al., 1997).

## Conclusion

The presence of enzymes in maternal tissues of the Galea spixii supported the idea that hormones produced by the maternal unit and fetus acted together for establishment and maintenance of pregnancy and embryo development. The Galea spixii embryo expressed CYP19 genes in post-implantation period, indicating they had the potential to produce steroid hormones.
